# Mixed Linkage β-1,3/1,4-Glucan Oligosaccharides Induce Defense Responses in *Hordeum vulgare* and *Arabidopsis thaliana*

**DOI:** 10.3389/fpls.2021.682439

**Published:** 2021-06-17

**Authors:** Sina Barghahn, Gregory Arnal, Namrata Jain, Elena Petutschnig, Harry Brumer, Volker Lipka

**Affiliations:** ^1^Department of Plant Cell Biology, Albrecht-von-Haller-Institute of Plant Sciences, The University of Göttingen, Göttingen, Germany; ^2^Michael Smith Laboratories, The University of British Columbia, Vancouver, BC, Canada; ^3^Department of Chemistry, The University of British Columbia, Vancouver, BC, Canada; ^4^Department of Biochemistry and Molecular Biology, The University of British Columbia, Vancouver, BC, Canada; ^5^Department of Botany, The University of British Columbia, Vancouver, BC, Canada

**Keywords:** DAMP-triggered immunity, MAMP-triggered immunity, elicitor, Arabidopsis, barley, β-1, 3/1, 4-mixed-linkage glucans

## Abstract

Plants detect conserved microbe-associated molecular patterns (MAMPs) and modified “self” molecules produced during pathogen infection [danger associated molecular patterns (DAMPs)] with plasma membrane-resident pattern recognition receptors (PRRs). PRR-mediated MAMP and/or DAMP perception activates signal transduction cascades, transcriptional reprogramming and plant immune responses collectively referred to as pattern-triggered immunity (PTI). Potential sources for MAMPs and DAMPs are microbial and plant cell walls, which are complex extracellular matrices composed of different carbohydrates and glycoproteins. Mixed linkage β-1,3/1,4-glucan (β-1,3/1,4-MLG) oligosaccharides are abundant components of monocot plant cell walls and are present in symbiotic, pathogenic and apathogenic fungi, oomycetes and bacteria, but have not been detected in the cell walls of dicot plant species so far. Here, we provide evidence that the monocot crop plant *H. vulgare* and the dicot *A. thaliana* can perceive β-1,3/1,4-MLG oligosaccharides and react with prototypical PTI responses. A collection of Arabidopsis innate immunity signaling mutants and >100 Arabidopsis ecotypes showed unaltered responses upon treatment with β-1,3/1,4-MLG oligosaccharides suggesting the employment of a so far unknown and highly conserved perception machinery. In conclusion, we postulate that β-1,3/1,4-MLG oligosaccharides have the dual capacity to act as immune-active DAMPs and/or MAMPs in monocot and dicot plant species.

## Introduction

Plants are constantly exposed to a variety of potential pathogens, including oomycetes, fungi and bacteria. To counteract potential intruders, plants have evolved a complex immune system. The presence of potentially harmful microbes can be recognized by plants through the perception of conserved microbe associated molecular patterns (MAMPs) that are absent from plants (Bigeard et al., 2015; Hückelhoven and Seidl, 2016). Besides the perception of these “non-self” molecules, plants are also able to sense altered plant-derived molecules that are only present upon pathogen attack or cell damage. These “self” molecules with eliciting activity are referred to as danger or damage associated molecular patterns (DAMPs) (Bigeard et al., 2015; Hou et al., 2019). The perception of MAMPs and DAMPs is mediated by plasma-membrane localized pattern recognition receptors (PRRs) that can be classified as receptor-like kinases (RLKs) or receptor-like proteins (RLPs) (Couto and Zipfel, 2016; Boutrot and Zipfel, 2017). Upon perception of MAMPs and DAMPs a signaling cascade is induced which results in the activation of pattern-triggered immunity (PTI). Typical PTI responses include intracellular Ca^2+^ elevation, generation of reactive oxygen species (ROS), phosphorylation of mitogen activated protein kinases (MAPKs) and transcriptional reprogramming (Bigeard et al., 2015; Hückelhoven and Seidl, 2016; Hou et al., 2019).

In recent years, various bacterial and fungal MAMPs as well as host-derived DAMPs of protein and carbohydrate origin have been identified (Saijo et al., 2018; Hou et al., 2019). The plant immune system is e.g., able to sense the two bacterial proteins flagellin and elongation factor TU (EF-Tu) as well as the plant-derived plant elicitor peptide 1 (PEP1) and its homologs (Gómez-Gómez et al., 1999; Gómez-Gómez and Boller, 2000; Kunze et al., 2004; Huffaker et al., 2006; Zipfel et al., 2006; Bartels et al., 2013). The homopolymer chitin and β-1,3-glucans derived from fungal and oomycete cell walls as well as peptidoglycan from bacterial cell walls represent carbohydrate-based MAMPs (Klarzynski et al., 2000; Aziz et al., 2003; Ménard et al., 2004; Kaku et al., 2006; Gust et al., 2007; Miya et al., 2007; Mélida et al., 2018). Interestingly, recent studies have revealed the carbohydrate-rich plant cell wall as source for several DAMPs. Oligosaccharides derived from the plant cell wall components homogalacturonan, cellulose, xyloglucan, mannan, and arabinoxylan have been identified as DAMP molecules (Aziz et al., 2007; Ferrari et al., 2013; Benedetti et al., 2015; Davidsson et al., 2017; de Azevedo Souza et al., 2017; Claverie et al., 2018; Locci et al., 2019; Zang et al., 2019; Mélida et al., 2020). These immunogenic cell wall fragments are likely to be released through the action of secreted microbial cell wall degrading enzymes (CWDEs) (Bacete et al., 2018).

While leucine-rich repeat (LRR) RLKs play a role in the perception of proteinaceous MAMPs and DAMPs, the wall-associated kinase 1 (WAK1) and lysin-motif (LysM) domain containing RLKs mediate perception of carbohydrate-based ligands (Brutus et al., 2010; Bigeard et al., 2015; Zipfel and Oldroyd, 2017). In Arabidopsis, chitin is detected by the LysM-RLKs CHITIN ELICITOR RECEPTOR KINASE 1 (CERK1), LYSIN MOTIF RECEPTOR KINASE 4 (LYK4) and LYK5 which form a heteromeric complex upon ligand perception (Kaku et al., 2006; Miya et al., 2007; Petutschnig et al., 2010; Liu et al., 2012; Wan et al., 2012; Cao et al., 2014; Erwig et al., 2017). Moreover, CERK1 was shown to be required for the perception of lipopolysaccharides (Gimenez-Ibanez et al., 2009; Desaki et al., 2018). CERK1 also mediates recognition of linear β-1,3-glucans as well as peptidoglycans together with the LysM RLPs LYSIN MOTIF DOMAIN-CONTAINING GLYCOSYLPHOSPHATIDYLINOSITOL-ANCHORED PROTEIN 1 (LYM1) and LYM3 (Willmann et al., 2011; Mélida et al., 2018; Wanke et al., 2020). However, the receptors involved in the perception of oligosaccharides derived from cellulose, xyloglucan, mannan, and arabinoxylan still remain elusive (de Azevedo Souza et al., 2017; Claverie et al., 2018; Locci et al., 2019; Zang et al., 2019; Mélida et al., 2020). Although several bacterial and fungal MAMPs, host-derived DAMPs and several cognate receptor (complexes) have been identified, it seems likely that plants are able to perceive more carbohydrate-based structures and that more receptors and co-receptors are involved in signal perception and transduction.

In contrast to the linear plant β-1,3- or β-1,4-glucose homopolymers callose and cellulose, mixed linkage β-1,3/1,4-glucans (β-1,3/1,4-MLGs) are linear polymers composed of glucosyl residues that are connected through both β-1,3- and β-1,4-glycosidic linkages (Burton and Fincher, 2009). In the plant kingdom, the presence of β-1,3/1,4-MLG is distributed asymmetrically. β-1,3/1,4-MLGs are found in the cell walls of most members of the monocot order *Poales* including the crop plant barley (*Hordeum vulgare*) as well as in evolutionary older plant lineages such as brown algae, liverworts and *Equisetum* spp., but are absent in dicot plants such as *Arabidopsis thaliana* (Zablackis et al., 1995; Popper and Fry, 2003; Fry et al., 2008; Sørensen et al., 2008; Burton and Fincher, 2009; Salmeán et al., 2017). Interestingly, β-1,3/1,4-MLGs have also been identified in lichen, namely *Cetaria islandica*, as well as in fungal, oomycete and bacterial species (Honegger and Haisch, 2001; Pettolino et al., 2009; Pérez-Mendoza et al., 2015; Rebaque et al., 2021). Thus, it is tempting to speculate that β-1,3/1,4-MLGs are more widespread cell wall components than previously thought.

As β-1,3/1,4-MLGs are present in the cell wall of monocotyledonous plants but are also abundant in bacterial and fungal species, they represent potential DAMPs and MAMPs in monocots and dicots, respectively. Thus, we analyzed the eliciting capacity of β-1,3/1,4-MLG oligosaccharides in *H. vulgare* and *A. thaliana* in this study. We found that β-1,3/1,4-MLG oligosaccharides derived from the hydrolysis of the *H. vulgare* β-1,3/1,4-MLG polysaccharide trigger canonical PTI responses in both, the monocot crop plant *H. vulgare* as well as the model dicot *A. thaliana*, suggesting a potential dual function as both DAMP and/or MAMP in a plant lineage-dependent manner. Reverse genetics and an accession screen in Arabidopsis revealed that known receptors and co-receptors of PTI are not involved in β-1,3/1,4-MLG oligosaccharide perception and that yet to be identified conserved molecular components mediate β-1,3/1,4-MLG oligosaccharide-induced signaling.

## Materials and Methods

### Plant Material and Growth Conditions

The Arabidopsis accession Col-0 was the background for all transgenic and mutant lines used in this study. Further Arabidopsis accessions that were used are listed in Supplementary Table 1. Seeds were surface sterilized by washing three times for 2 min with 70% EtOH and 0.05% Tween-20 with agitation. Seeds were afterward washed two times for 1 min with 100% EtOH and dried. The dry seeds were either sown on soil or grown on aqueous ½ Murashige and Skoog (MS) medium. For RNA extraction and MAPK experiments, 7-days old seedlings were transferred into individual wells (two seedlings per well) of a transparent 24-well plate and grown for seven further days. Plants were grown in a growth cabinet (CLF Plant Climatics, Wertingen, Germany) under short day conditions (12 h light, 12 h darkness).

*H. vulgare* was grown on soil in a growth chamber (Johnson Controls, Milwaukee, WI, United States) with long day conditions (16 h light, 26°C, 200 m^–2^ s^–1^, 65% relative humidity).

### Elicitors

Polymeric chitin of shrimp shells was obtained from Sigma-Aldrich (C9752-5G, Sigma), oligogalacturonides (OGs) were kindly provided by Simone Ferrari (Sapienza University of Rome) and the flg22 peptide (Felix et al., 1999) was synthesized by Thermo Scientific at a purity level of 98%. β-1,3/1,4-MLG tetra- and -trisaccharides used in this study were obtained from Megazyme (O-BGTETB, O-BGTETC, O-BGTRIA, and O-BGTRIB) and dissolved in ultrapure water at a concentration of 10 mg ml^–1^. Fourteen-day-old Arabidopsis seedlings or leaf discs from second leaves of 14-days old *H. vulgare* plants were treated with elicitors added to liquid ½ MS plus sucrose medium. Unless otherwise stated, 100 μg ml^–1^ chitin, 100 nM flg22, 150 μM, or 190 μM of the β-1,3/1,4-MLG tetra- and -trisaccharides, were used for calcium and ROS burst assays, 10 μg ml^–1^ chitin, 10 μg ml^–1^ OGs, 50 nM flg22, 15 μM, or 10 μM of the β-1,3/1,4-MLG tetra- and -trisaccharides, were used for MAPK experiments and gene expression analyses.

To generate *H. vulgare* -derived β-1,3/1,4-MLG oligosaccharides, 10 mg ml^–1^ *H. vulgare* β-1,3/1,4-MLG polysaccharide (P-BGBL, Megazyme) was dissolved in 100 mM sodium phosphate buffer (pH = 6.5). 0.025 or 1 U ml^–1^ lichenase (E-LICHN, Megazyme) was added to the solution and incubated for different times at 40°C with agitation. The hydrolysis was stopped by incubation in boiling water for at least 15 min. The hydrolysis products were tested via Thin Layer Chromatography (TLC). 5 μl of the analytes were applied onto a TLC Silica gel plate (TLC silica gel 60, 1.05721.0001, Merck). Upon drying, the plate was put into running buffer (isopropanol:ethylacetate:water, 2:2:1). Upon drying of the plate, the plate was wetted with a TLC staining solution (10% sulfuric acid in methanol) and incubated on a heating plate at 99°C. Seedlings were treated with a 1:10 dilution of the hydrolysis products.

### Calcium Measurements

Intracellular calcium was measured using an aequorin-based calcium assay (Ranf et al., 2012). Calcium responses in the absence of an elicitor was included as negative control. The Ca^2+^ concentrations were calculated and normalized according to Rentel and Knight, 2004 and are depicted as L/Lmax with L representing the luminescence at any time point upon β-1,3/1,4-MLG oligosaccharide or MAMP treatment and Lmax representing the total remaining aequorin. To calculate Lmax, the luminescence obtained upon treatment with the discharge solution was integrated.

### ROS Measurements

The generation of ROS was determined using a luminol-based assay. Leaf discs (4 mm diameter) of 5-7 weeks old Arabidopsis plants or 10–12 days old *H. vulgare* plants were incubated in water overnight in a flat-bottom 96-well plate. The water was replaced with a luminol solution [10 μg ml^–1^ Horseradish peroxidase (P6782, Sigma), 100 μM L-012 (120-04891, WAKO Chemicals)] containing no elicitor, elicitors at the indicated concentrations or a 1:10 dilution of *H. vulgare* β-1,3/1,4-MLG polysaccharide hydrolysis products. Luminescence was recorded with a TECAN infinite^®^ M200 plate reader for 60 min in 1 min intervals with an integration time of 150 ms.

### MAPK Assays

Arabidopsis seedlings were grown *in vitro* as described above. One day before the treatment, the medium was replaced with 500 μl ½ fresh MS medium to ensure equal volumes. 14-days old seedlings were treated with the elicitors for 12 min and directly frozen in liquid nitrogen. *H. vulgare* plants were grown on soil as described above, 12–14 leaf discs of 4 mm diameter were harvested from second leaves of 14-days old plants and incubated for 16 h in 2 ml ultrapure water. The leaf discs were transferred to fresh ultrapure water and incubated for 30 min. Subsequently, elicitor solutions were added to the indicated final concentrations. Negative control samples were treated with an equivalent volume of water. The leaf discs were incubated for 12 min and then directly frozen in liquid nitrogen. The frozen seedlings or leaf discs were homogenized in 600 or 200 μl extraction buffer, respectively (Petutschnig et al., 2010). After centrifugation for 10 min at 4°C at 13.000 rpm, the protein concentration was determined via Bradford Assay with BSA as standard and protein concentrations were equalized. Protein extracts were frozen at −20°C. Samples were separated on a 10% SDS gel. Immunoblot analysis were performed using Phospho p44/42 (#9101, Cell Signaling Technology, 1:5000) as primary antibody and a goat anti-rabbit IgG (A3687, 1:5000, Sigma Aldrich) as secondary antibody.

### RNA Isolation and qRT-PCR

**FIGURE 1:**
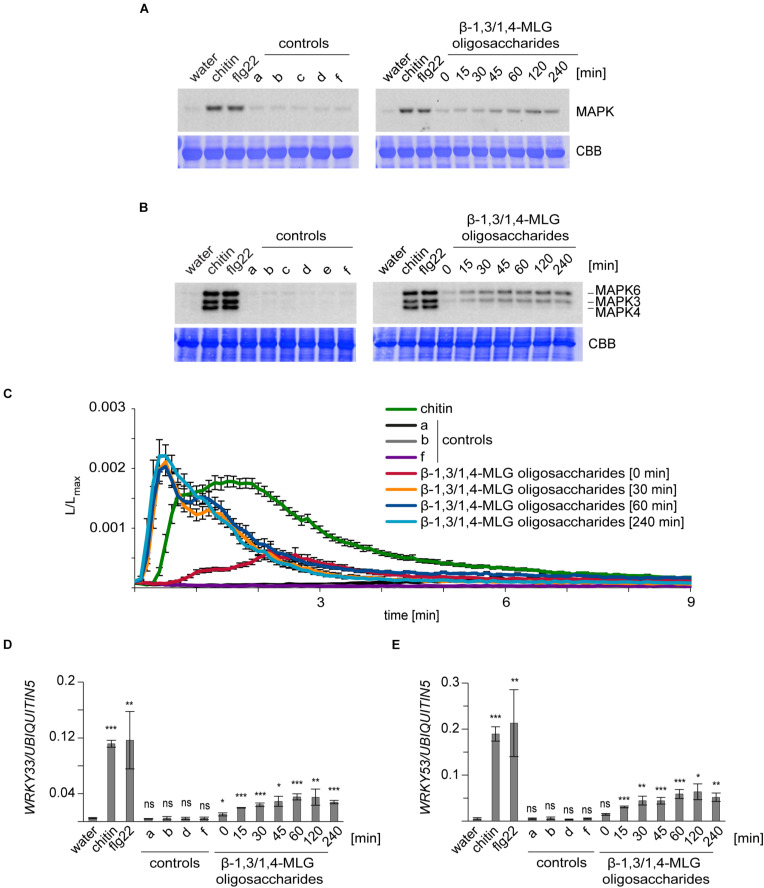
Enzymatically-generated β-1,3/1,4-MLG oligosaccharides induce immune responses in *A. thaliana* and *H. vulgare*. β-1,3/1,4-MLG oligosaccharides were generated by incubation of the β-1,3/1,4-MLG polysaccharide (10 mg ml^–1^) with the *B. subtilis* lichenase (0.025 U/ml) and was stopped at several time points (0, 15, 30, 45, 60, 120, or 240 min). For the treatment, a 1:10 dilution of the hydrolysis products was used. Chitin (10 μg ml^–1^ for MAPK activation and defense gene expression; 100 μg ml^–1^ for calcium elevation) and flg22 (50 nM) served as positive controls. As negative controls, water, sodium phosphate buffer (a, 10 mM), β-1,3/1,4-MLG polysaccharide (b, 1 mg ml^–1^), active lichenase (c, 0.0025 U ml^–1^), heat-inactivated lichenase (d, 0.0025 U ml^–1^; e, 0.00125 U ml^–1^) and β-1,3/1,4-MLG polysaccharide (1 mg ml^–1^) and heat-inactivated lichenase (f, 0.0025 U ml^–1^) were used. **(A,B)**: MAPK phosphorylation in **(A)** leaf discs of 12–14 day-old *H. vulgare* plants and **(B)** 14-day-old Arabidopsis Col-0 seedlings upon elicitation with β-1,3/1,4-MLG oligosaccharides. As loading control, the PVDF membrane was stained with Coomassie Brilliant Blue (CBB). The data shown are derived from one of three biological replicates that gave similar results. **(C)** Calcium elevation in 8–10 day-old Arabidopsis Col-0 aequorin lines in response to enzymatically generated β-1,3/1,4-MLG oligosaccharides. The luminescence was assessed immediately after elicitor treatment (L) and was normalized to total luminescence measured upon addition of calcium (L_*max*_). The data show mean of twelve seedlings with SEM from one of three biological experiments that produced similar results. **(D,E)**: Expression of the defense genes *WRKY33* **(D)** and *WRKY53* **(E)** in 14-day-old Arabidopsis Col-0 seedlings upon treatment with enzymatically generated β-1,3/1,4-MLG oligosaccharides. Data are presented as means of three biological replicates (with three technical replicates each) ± SD. *UBIQUITIN5* was used as reference gene. Statistical significance (unpaired student’s *t*-test) of elicitor treatment compared to water treatment: ns = *p* > 0.5; * = *p* ≤ 0.5, ** = *p* ≤ 0.05, *** = *p* ≤ 0.001.

**FIGURE 2:**
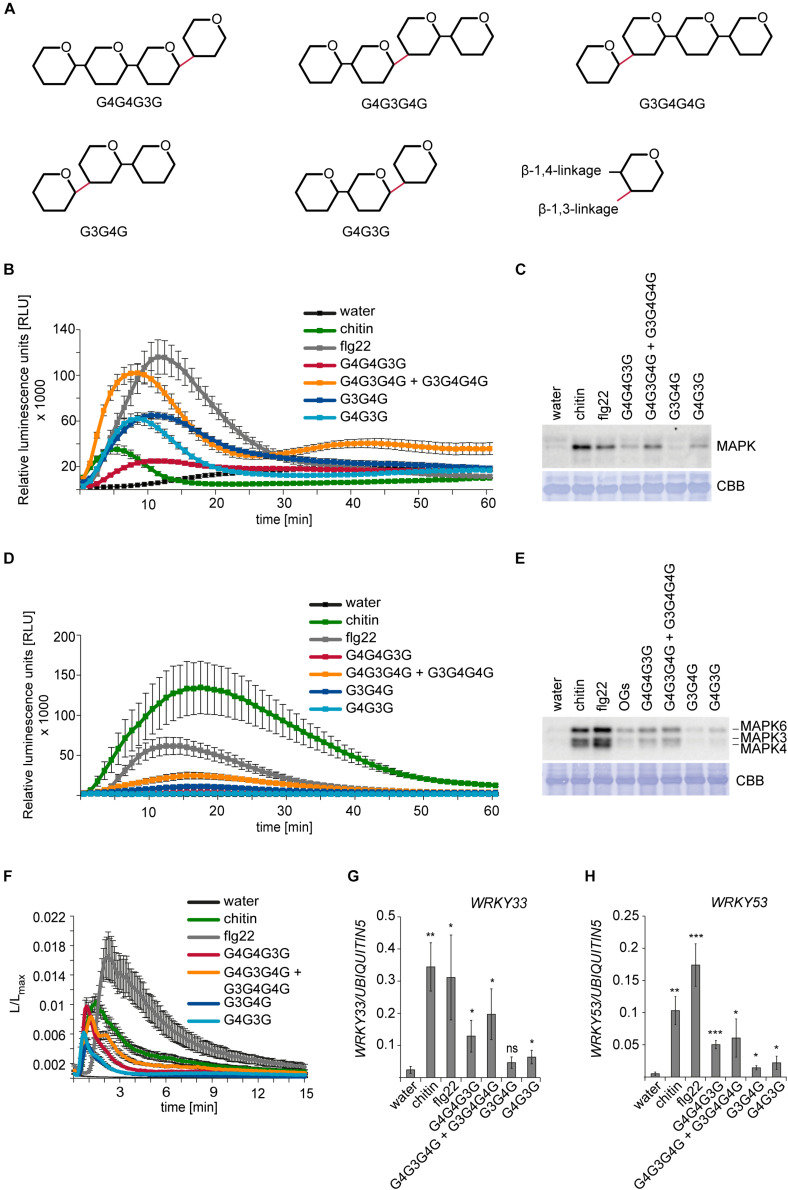
Activation of PTI in *H. vulgare* and *A. thaliana* by β-1,3/1,4-MLG tetrasaccharides and trisaccharides. For ROS production and calcium elevation, 150 μM of the β-1,3/1,4-MLG tetrasaccharides and 190 μM of the β-1,3/1,4-MLG trisaccharides were used. Water, chitin (100 μg ml^– 1^) and flg22 (100 nM) served as controls. For MAPK activation and calcium elevation, 15 μM and 19 μM of the β-1,3/1,4-MLG tetrasaccharides and β-1,3/1,4-MLG trisaccharides were used, respectively. As controls, water, chitin (10 μg ml^–1^) and flg22 (50 nM) and oligogalacturonides (OG; 10 μg ml^–1^) were used. **(A)** Structure of the β-1,3/1,4-MLG MLG tetrasaccharides and -trisaccharides used in the experiments. **(B)** ROS production in leaf discs of 12–14-days old *H. vulgare* plants upon elicitation with β-1,3/1,4-MLG tetrasaccharides and trisaccharides. Data represent the mean of eight leaf discs from one out of three biological replicates that gave similar results. Error bars represent SEM. **(C)** Phosphorylation of MAPK in leaf discs of 14-day old *H. vulgare* plants in response to β-1,3/1,4-MLG tetrasaccharides and -trisaccharides. As loading control, the PVDF membrane was stained with Coomassie Brilliant Blue (CBB). Data show one biological replicate. The experiment was repeated three times with similar results. **(D)** Generation of ROS in Arabidopsis leaf discs of 5–7 weeks old Arabidopsis Col-0 plants upon treatment with β-1,3/1,4-MLG tetra- and trisaccharides. Data shown the mean of eight leaf discs from one biological replicate. Error bars represent SEM. The experiment was repeated three times with similar results. **(E)** MAPK activation in 14-day old Arabidopsis Col-0 seedlings upon elicitation with β-1,3/1,4-MLG tetra- and trisaccharides. As loading control, the PVDF membrane was stained with Coomassie Brilliant Blue (CBB). Data show the result of one out of three biological replicated that showed similar results. **(F)** Calcium elevation in 8–10 day-old Arabidopsis Col-0 seedlings expressing aequorin in response to β-1,3/1,4-MLG tetra- and trisaccharides. The luminescence was assessed immediately after elicitor treatment (L) and was normalized to total luminescence upon addition of calcium (L_*max*_). The data show means of twelve seedlings + SEM from one of three biological experiments that gave similar results. **(G,H)** Expression of the defense genes *WRKY33* **(G)** and *WRKY53* **(H)** in 14-days old Arabidopsis Col-0 seedlings upon treatment with β-1,3/1,4-MLG tetra- and trisaccharides. Data are presented as means of three biological replicates (with three technical replicates each) ± SD. *UBIQUITIN5* was used as reference gene. Statistical significance (unpaired student’s *t*-test) of elicitor treatment compared to water treatment: ns = *p* > 0.5; * = *p* ≤ 0.5, ** = *p* ≤ 0.05, *** = *p* ≤ 0.001.

Arabidopsis seedlings were grown *in vitro* as described above. One day before the treatment, the medium was replaced with 500 μl ½ MS medium to ensure equal volumes. 14-day-old seedlings were treated with the elicitors for 30 min and directly frozen in liquid nitrogen. Total RNA was extracted from seedlings using Qiazol (Qiagen, Hilden, Germany) and digested with DNase (EN0521, Thermo Scientific). 1 μg RNA per 20 μl reaction were used to generate cDNA using RevertAid^TM^ H Minus M-MulVRT (EP0451, Thermo Scientific). For qRT-PCRs, 3 μL of 1:500 diluted cDNA was used to analyze gene expression with SsoFast EvaGreen supermix (1725204, BioRad) using the following PCR conditions: 95°C for 30 s, 45 cycles of 95°C for 5 s, and 55°C for 10 s, followed by fluorescence reading. For normalization, *UBIQUITIN5* was amplified in parallel on each plate. Aliquots of cDNAs used within one experiment were pooled and a dilution series was prepared from the pool to calculate primer efficiencies. Primer pair efficiencies were determined to be 98.53% (*WRKY33*), 100.06% (*WRKY53*) and 103.58% (*Ubiquitin5*) for the experiment shown in Figures 1D,E and 92.91% (*WRKY33*), 96.56% (*WRKY53*) and 104.82% (*Ubiquitin5*) for the experiment shown in Figures 2G,H. Melting curves and no-template controls were analyzed to rule out primer-dimer formation and contaminations. Primers are listed in Supplementary Table 2.

### Seedling Growth Inhibition

Arabidopsis seedlings were grown for 5 days on ½ MS medium and then transferred to 24-well plates (one seedling per well). Each well contained 500 μL ½ MS medium with either no elicitor, or one of the following substances:1 μM flg22, 10 mM Sodium Phosphate buffer (pH = 6.5) or a 1:10 dilution of the enzymatically generated *H. vulgare* β-1,3/1,4-MLG oligosaccharides. Pictures were taken of 13-day-old seedlings. To determine the dry weight, seedlings were dried for 1 day at 65°C and the total weight of all eight seedlings was determined.

### Carbohydrate Analysis

High performance anion exchange chromatography with pulsed amperometric detection (HPAEC-PAD) was performed using a Dionex ICS-5000 HPLC system equipped with an AS-AP autosampler in a sequential injection configuration using the Chromeleon software version 7. 10 μl of the samples were injected on a 3 × 250 mm Dionex Carbopac PA200 column (Thermo Scientific, Waltham, United States). 56 μM of the β-1,3/1,4-MLG tetrasaccharide or 45 μM of the β-1,3/1,4-MLG trisaccharide were loaded onto the column. The gradient was used as follows: 0–5 min, 10% B, 3.5% C (initial conditions); 5–12 min 10% B, linear gradient from 0–30% C; 12.0–12.1 min, 50% B, 50% C; 12.1–13.0 min, exponential gradient of B and C, back to initial conditions, 13–17 min initial conditions. Solvent A was ultrapure water, solvent B was 1 M sodium hydroxide and solvent C was 1 M sodium acetate.

Matrix Assisted Laser Desorption Ionization–Time of Flight (MALDI-TOF) analysis of mixed-linkage glucans was performed with a Bruker Autoflex system (Bruker Daltonics) operated in reflectron mode. 10 mg/ml of the oligosaccharide were mixed 1:5 with 2,5-dihiydroxybenzoic acid in 1:1 H_2_O:MeOH on a Bruker MTP 384 grounded steel MALDI plate. The samples were allowed to dry and directly analyzed.

### Accession Numbers

The Arabidopsis mutant lines used in this study were: *cerk1*-2 (GABI_096F09), *lyk5*-2 *lyk4*-2 (SALK_131911C × GABI_897A10), *lym2*-1 (SAIL_343B03), *lym2*-4 (GABI-Kat 165 H02), *efr*-1 (SALK_044334), *fls2c* (SAIL_691_C4), *bak1*-4 (SALK_116202), *bak1*-5 (Schwessinger et al., 2011), *sobir1*-12 (SALK_050715), and *sobir1*-14 (GABI-Kat_643F07).

## Results

### Enzymatically Generated β-1,3/1,4-MLG Oligosaccharides Induce Immune Responses in *H. vulgare* and *A. thaliana*

To test the potential MAMP or DAMP activities of β-1,3/1,4-MLG polymer and enzymatically derived oligosaccharides in the monocot and dicot model plants *H. vulgare* and *A. thaliana*, we first used commercially available *H. vulgare* β-1,3/1,4-MLG polysaccharide that we incubated with *Bacillus subtilis* lichenase (Supplementary Figure 1). *H. vulgare* β-1,3/1,4-MLG is composed of β-1,3-linked cellotriosyl or cellotetrasyl units (Burton and Fincher, 2014) and *B. subtilis* lichenase hydrolyses β-1,4-bonds that immediately follow β-1,3-linkages in β-1,3/1,4-MLG polymers (Planas, 2000). Thus, enzymatic end products are *H. vulgare* β-1,3/1,4-MLG tri- and tetrasaccharides such as G4G3G and G3G4G4G, where G represents glucose and 3 and 4 indicate the β-1,3- and β-1,4-linkages, respectively. We stopped lichenase-mediated hydrolysis at different incubation times (0, 15, 30, 45, 60, 120, and 240 min), to obtain hydrolyzates containing β-1,3/1,4-MLG oligosaccharides with different degrees of polymerization (DP). The hydrolysis of the β-1,3/1,4-MLG polysaccharide was confirmed with TLC and revealed an increase in abundance of β-1,3/1,4-MLG oligosaccharides with a shorter DP over time (Supplementary Figure 1).

The activation of MAPKs upon MAMP or DAMP perception is a conserved immune response in *H. vulgare* and *A. thaliana* (Bigeard et al., 2015; Hückelhoven and Seidl, 2016), which can be monitored by Western blot analysis (Figures 1A,B). Our experiments revealed that only enzymatically generated β-1,3/1,4-MLG oligosaccharides induced the phosphorylation of MAPKs in *H. vulgare* and *A. thaliana*, whereas application of the β-1,3/1,4-MLG polysaccharide, lichenase enzyme and mock controls did not trigger MAPK activation (Figures 1A,B). Interestingly, only MAPK6 and MAPK3 but not MAPK4/11 were phosphorylated upon elicitation with β-1,3/1,4-MLG oligosaccharides in *A. thaliana* (Figure 1B). Next, we used transgenic Arabidopsis plants producing the Ca^2+^-sensor aequorin to analyze treatment-dependent influx of calcium (Figure 1C), another MAMP/DAMP-induced immune response (Bigeard et al., 2015). Again, only enzymatically generated hydrolysis products of the β-1,3/1,4-MLG polysaccharides, but not the β-1,3/1,4-MLG polysaccharide led to a fast and transient influx of Ca^2+^ which peaked at 30 s and lasted for about 3 min (Figure 1C). Finally, the expression of the two transcription factors *WRKY33* and *WRKY53*, which were previously shown to be up-regulated in response to MAMPs (Cao et al., 2014), was analyzed by qRT-PCR. The expression of both, *WRKY33* and *WRKY53*, was upregulated upon β-1,3/1,4-MLG oligosaccharides elicitation (Figures 1D,E). Notably, the intensity of the tested immune responses in *A. thaliana* and *H. vulgare* was more pronounced upon treatment with hydrolyzates containing a high amount of β-1,3/1,4-MLG oligosaccharides with a short DP (60, 120, or 240 min incubation time, Figure 1 and Supplementary Figure 1). These results indicate that lichenase-generated β-1,3/1,4-MLG oligosaccharides can be perceived by *H. vulgare* and *A. thaliana* and induce canonical pattern-triggered immune responses.

### β-1,3/1,4-MLG Tetra- and Trisaccharides Activate PTI Responses

To confirm the ability of β-1,3/1,4-MLG oligosaccharides to trigger innate immune responses in the monocot and dicot models *H. vulgare* and *A. thaliana*, commercially available β-1,3/1,4-MLG preparations with defined linkage sequences were tested for MAMP/DAMP activity: a β-1,3/1,4-MLG tetrasaccharide (G4G4G3G), a β-1,3/1,4-MLG tetrasaccharide mixture (G4G3G4G + G3G4G4G), as well as two β-1,3/1,4-MLG trisaccharides (G3G4G and G4G3G) (Figure 2A). The purity and the masses of the obtained β-1,3/1,4-MLG oligosaccharides were confirmed using HPAEC-PAD and MALDI-TOF (Supplementary Figure 2).

First, we tested the generation of ROS, which represents another canonical and early MAMP/DAMP-inducible plant response (Bigeard et al., 2015; Hückelhoven and Seidl, 2016). In *H. vulgare*, ROS production was induced upon treatment with the β-1,3/1,4-MLG tetrasaccharide mixture and the two β-1,3/1,4-MLG trisaccharides. The generation of ROS in response to β-1,3/1,4-MLG oligosaccharides was weaker than the flagellin (flg22)-induced ROS burst, but stronger than the chitin-induced ROS burst (Figure 2B). Phosphorylation of MAPK in *H. vulgare* was also induced in response to the β-1,3/1,4-MLG tetrasaccharide mixture and the β-1,3/1,4-MLG trisaccharide G4G3G, whereas it was almost or completely undetectable upon G4G4G3G or G3G4G treatment (Figure 2C). In *A. thaliana* Col-0 plants, only slight generation of ROS could be detected upon treatment with the β-1,3/1,4-MLG tetrasaccharide mixture, which was weaker than chitin or flg22-induced ROS production (Figure 2D). The phosphorylation of MAPK6 and MAPK3 was triggered upon application of β-1,3/1,4-MLG tetrasaccharides but was almost undetectable in response to β-1,3/1,4-MLG trisaccharides (Figure 2E). The influx of calcium ions was induced in response to all commercially available β-1,3/1,4-MLG tetra- and trisaccharides (Figure 2F). Furthermore, analysis of the expression of *WRKY33* and *WRKY53* revealed a transcriptional up-regulation of both genes upon β-1,3/1,4-MLG tetra- and trisaccharide elicitation (Figures 2G,H).

Together, these experiments corroborate the ability of β-1,3/1,4-MLG oligosaccharides to act as elicitors of immune responses in the monocot *H. vulgare* and the dicot *A. thaliana*. Notably, the analyzed immune responses were stronger in response to β-1,3/1,4-MLG tetrasaccharides in both *H. vulgare* and *A. thaliana*, possibly indicating that the corresponding receptor has a higher affinity to longer β-1,3/1,4-MLG oligosaccharides.

### Known Receptors and co-Receptors Are Not Involved in Perception of β-1,3/1,4-MLG Oligosaccharides

**FIGURE 3:**
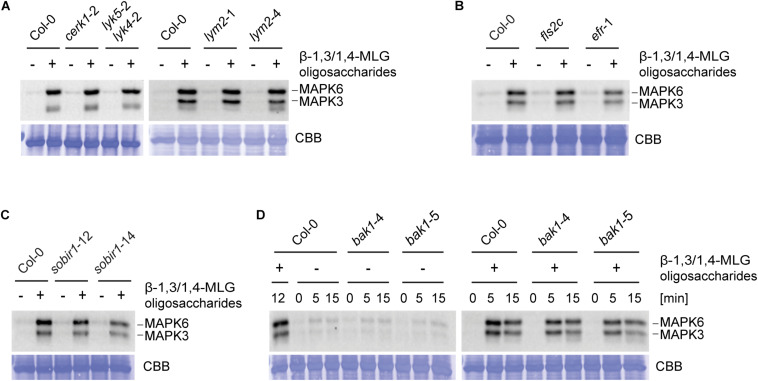
β-1,3/1,4-MLG oligosaccharides induced activation of MAPK is independent of known PTI components. MAPK activation in 14-day old seedlings of **(A)** Col-0, *cerk1*-2, *lyk5*-2 *lyk4*-2, *lym2*-1, and *lym2*-4, **(B)** Col-0, *fls2c*, *efr*-1, **(C)** Col-0, *sobir1*-12, *sobir1*-14 and **(D)** Col-0, *bak1*-4 and *bak1*-5 upon elicitation with enzymatically generated with β-1,3/1,4-MLG oligosaccharides. β-1,3/1,4-MLG oligosaccharides were generated upon incubation of the MLG polymer (10 mg ml^–1^) with *B. subtilis* lichenase (1 U ml^–1^) for 60 min (Supplementary Figure 3). For seedling treatment, a 1:10 dilution of the hydrolysis products was used. Sodium phosphate buffer (1 mM) served as negative control. **(A–C)** Western Blot shows MAPK phosphorylation upon elicitation with the indicated elicitors for 12 min. **(D)** Western Blot shows phosphorylated MAPK at different time points (0, 5, and 15 min). As loading control, the PVDF membrane was stained with Coomassie Brilliant Blue (CBB). Data show the result from one biological replicate. The experiments were repeated twice with similar results.

To identify molecular components involved in β-1,3/1,4-MLG oligosaccharide perception and signaling, only the dicot model plant *A. thaliana* was used, as a substantial number of mutants of components of the plant immune system are available. LysM domain containing RLKs have previously been shown to be involved in the perception of carbohydrate-based elicitors (Miya et al., 2007; Shimizu et al., 2010; Willmann et al., 2011; Wan et al., 2012; Cao et al., 2014; Desaki et al., 2018; Mélida et al., 2018; Wanke et al., 2020). The Arabidopsis genome encodes for five LysM-RLKs, namely CERK1/LYK1, and LYK2 to LYK5 (Zhang et al., 2007). CERK1 is involved in the detection of chitin together with LYK4 and LYK5 as well as bacterial peptidoglycan and β-1,3-glucans (Kaku et al., 2006; Miya et al., 2007; Petutschnig et al., 2010; Willmann et al., 2011; Liu et al., 2012; Cao et al., 2014; Erwig et al., 2017; Mélida et al., 2018). The LysM-RLP LYM2 does not play a role in canonical chitin-triggered immune responses, but facilitates the reduction of molecular flux via plasmodesmata in response to chitin (Faulkner et al., 2013; Cheval et al., 2020). To test a potential involvement of CERK1, LYK5, LYK4, and LYM2 in perception of β-1,3/1,4-MLG oligosaccharides, MAPK phosphorylation in *cerk1*-2, *lyk5*-2 *lyk4*-2, *lym2*-1, and *lym2*-4 upon elicitation with lichenase-generated β-1,3/1,4-MLG oligosaccharides was assessed. To generate β-1,3/1,4-MLG oligosaccharides, *H. vulgare* derived β-1,3/1,4-MLG polysaccharide was incubated for 60 min with *B. subtilis* lichenase. The hydrolysis was confirmed via TLC (Supplementary Figure 3). The intensity of MAPK phosphorylation was not altered in the tested mutants in comparison to the wild-type (Figure 3A) indicating that perception of β-1,3/1,4-MLG oligosaccharides is independent of CERK1, LYK4, LYK5, and LYM2.

The LRR-RLK FLAGELLIN SENSING 2 (FLS2) and the LRR-RLK EF-TU RECEPTOR (EFR) are required for the perception of the prototypical protein-derived MAMPs flg22 and elf18, respectively (Gómez-Gómez and Boller, 2000; Zipfel et al., 2006). In order to assess whether EFR or FLS2 are involved in β-1,3/1,4-MLG oligosaccharide perception, phosphorylation of MAPK in *fls2c* and *efr-1* in response to enzymatically generated β-1,3/1,4-MLG oligosaccharides (Supplementary Figure 3) was tested. Western Blotting revealed the same intensity of MAPK phosphorylation in Col-0, *fls2c*, and *efr-1*, suggesting that neither FLS2 nor EFR play a role in β-1,3/1,4-MLG oligosaccharides perception (Figure 3B).

The co-receptors BRASSINOSTEROID INSENSITIVE 1-associated receptor kinase 1 (BAK1) and the adaptor kinase SUPPRESSOR OF BRASSINOSTEROID INSENSITIVE 1 (SOBIR1) are required for the activation of early immune responses upon perception of several elicitors (Chinchilla et al., 2006; Heese et al., 2007; Roux et al., 2011; Schwessinger et al., 2011). To address whether BAK1 or SOBIR1 are required for β-1,3/1,4-MLG oligosaccharide perception, MAPK phosphorylation in response to β-1,3/1,4-MLG oligosaccharides was monitored in mutants of SOBIR1 and BAK1. The level of MAPK phosphorylation was similar between the wild-type (Col-0) and the tested mutants, suggesting that neither SOBIR1 nor BAK1 are involved in perception of β-1,3/1,4-MLG oligosaccharides (Figures 3C,D). Together, these results demonstrate that the tested prototypical receptors and co-receptors are dispensable for perception of β-1,3/1,4-MLG oligosaccharides and prompt the conclusion that a yet unknown receptor (complex) mediates β-1,3/1,4-MLG oligosaccharide perception and signal transduction.

### Arabidopsis Ecotypes Exhibit a Conserved Capacity for β-1,3/1,4-MLG Oligosaccharide Perception

As PTI can vary between different Arabidopsis accessions due to distinct genomic receptor repertoires, pattern-sensitive and pattern-insensitive accessions have previously been used to identify PRRs governing perception via comparative genomics (Gómez-Gómez et al., 1999; Gómez-Gómez and Boller, 2000; Jehle et al., 2013; Zhang et al., 2013).

**FIGURE 4:**
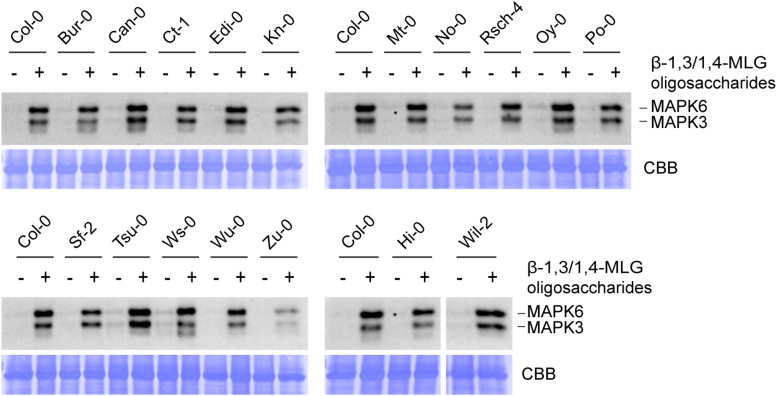
Lichenase-generated β-1,3/1,4-MLG oligosaccharides induce MAPK activation in different Arabidopsis accessions. MAPK activation in 14-day-old seedlings of various ecotypes in response to enzymatically generated β-1,3/1,4-MLG oligosaccharides. β-1,3/1,4-MLG oligosaccharides were generated upon incubation of the MLG polymer (10 mg ml^–1^) with the *B. subtilis* lichenase (1 U ml^–1^) for 60 min (Supplementary Figure 3). For the treatment, a 1:10 dilution of the hydrolysis products was used. Sodium phosphate buffer (1 mM) served as negative control. As loading control, the PVDF membrane was stained with Coomassie Brilliant Blue (CBB). Data show the result from one biological replicate.

To identify potential Arabidopsis ecotypes that are insensitive toward β-1,3/1,4-MLG oligosaccharides, the level of MAPK phosphorylation upon application of β-1,3/1,4-MLG oligosaccharides was determined in 112 different accessions. First, parental ecotypes of the Multiparent Advanced Generation Inter-Cross (MAGIC) recombinant inbred lines (Kover et al., 2009) were chosen, because these would have facilitated mapping of potential β-1,3/1,4-MLG oligosaccharide receptors. However, all parental ecotypes responded to β-1,3/1,4-MLG oligosaccharide treatment with an almost invariant MAPK activation pattern (Figure 4). Similarly, a collection of 95 additional Arabidopsis accessions that were tested for their ability to perceive β-1,3/1,4-MLG-derived oligosaccharides as MAMP, also showed qualitatively unaltered MAPK phosphorylation capacity in response to enzymatically produced β-1,3/1,4-MLG oligosaccharides (Supplementary Figure 4). The fact that more than 100 Arabidopsis accessions were sensitive toward β-1,3/1,4-MLG oligosaccharides suggest that the underlying perception and signaling machinery is highly conserved within the species *Arabidopsis thaliana*.

## Discussion

During plant-microbe interactions, microbial and plant CWDEs are secreted into the extracellular space, where they act on their opponents’ cell walls and release small oligosaccharides (Bacete et al., 2018). Thus, the carbohydrate-rich cell walls of plants and their pathogens represent a source for potential DAMPs and MAMPs. In the last years, several cell wall-derived DAMPs and MAMPs have been identified (Saijo et al., 2018; Hou et al., 2019; Pontiggia et al., 2020), but it is conceivable that a substantially larger number of cell wall-derived ligands can be perceived by plants. Here we provide evidence that β-1,3/1,4-MLG oligosaccharides activate immune responses in more than 100 accessions of the dicot model plant *A. thaliana* and the monocot crop plant *H. vulgare*.

The β-1,3/1,4-MLG polysaccharide can be found in the cell wall of most members of the *Poales* including *H. vulgare*, but also in evolutionary older plant lineages, e.g., brown algae or horsetail (Fry et al., 2008; Burton and Fincher, 2009; Salmeán et al., 2017). Thus, β-1,3/1,4-MLG oligosaccharides can be classified as DAMPs in monocot species. The cell wall derived OGs are DAMPs that are released upon partial degradation of the pectin component homogalacturonan by fungal polygalacturonases (Ferrari et al., 2013; Benedetti et al., 2015; Pontiggia et al., 2020). Similarly, β-1,3/1,4-MLG oligosaccharides are likely to be released from the abundant monocot β-1,3/1,4-MLG polysaccharide through enzymatic activity of microbial glucanases secreted during monocot plant-microbe interactions.

Genomes of biotrophic, hemibiotrophic and necrotrophic plant pathogens contain a high number of CWDEs of which only a few have been biochemically characterized (Kubicek et al., 2014; Zhao et al., 2014). The genome of the biotrophic barley powdery mildew *Blumeria graminis* f.sp. *hordei* (*Bgh*) was shown to encode for 67 glycoside hydrolases (GHs) that can be classified into 25 different families including e.g., GH5, GH16 or GH17 (Zhao et al., 2014). Enzymes classified as GH5, GH16 or GH17 are predicted to catalyze the hydrolysis of cellulose, hemicelluloses, β-1,3-glucans and β-1,3/1,4-MLGs (Carbohydrate Active Enzymes database^1^; Lombard et al., 2014; Zhao et al., 2014), however, none of the *Bgh* GHs have been biochemically characterized. Notably, transcriptome analysis showed that the expression of one GH16 enzyme (*Bgh01441*) and other CWDEs of barley powdery mildew are upregulated during infection of immunocompromised Arabidopsis mutants, suggesting that they may be involved in degradation of the plant cell wall (Hacquard et al., 2013). The analysis of plant pathogen secretomes and the biochemical characterization of identified CWDEs have the potential to reveal whether CWDEs acting on the β-1,3/1,4-MLG polysaccharide are present in a given monocot plant pathogen species.

In contrast to monocot species, dicot plants such as *A. thaliana* do not contain β-1,3/1,4-MLGs in their cell walls (Zablackis et al., 1995; Burton and Fincher, 2009). However, the experimental data of this work and a recently published study (Rebaque et al., 2021) clearly demonstrate that Arabidopsis plants can detect β-1,3/1,4-MLG oligosaccharides and react with canonical PTI responses. As β-1,3/1,4-MLG oligosaccharides are not present in Arabidopsis, they are likely to function as a MAMP in this species. Interestingly, β-1,3/1,4-MLG polysaccharides have previously been identified in the plant pathogenic fungus *Rhynchosporium secalis*, the oomycete *Hyaloperonospera arabidopsidis* and the endosymbiotic bacterium *Sinorhizobium meliloti* (Pettolino et al., 2009; Pérez-Mendoza et al., 2015; Rebaque et al., 2021). Microbial cell walls can contain between 50–60% glucans (Bowman and Free, 2006), however, to what extent β-1,3/1,4-MLGs contribute to their chemical structure remains largely elusive. Thus, β-1,3/1,4-MLGs may be more abundant microbial cell wall components than previously thought.

Upon pathogen attack, plant chitinases and β-1,3-glucanases are secreted, act on fungal cell wall components and release immunogenic oligosaccharides (Keen and Yoshikawa, 1983; Ham, 1991; Uknes et al., 1992; Doxey et al., 2007; Balasubramanian et al., 2012; Pusztahelyi, 2018). Similarly, β-1,3;1,4-glucanases may be secreted by host and non-host plants. The Arabidopsis genome harbors more than 400 genes encoding for GHs, of which only a few have been characterized (Carbohydrate Active Enzymes database (see text footnote 1); Lombard et al., 2014). Glucanases that may act on β-1,3/1,4-MLGs can be found in GH families 5, 6, 7, 8, 9, 11, 12, 16, and 17. The Arabidopsis genome harbors 13, 26, 33, and 51 genes encoding for enzymes categorized as GH5, GH9, GH16, and GH17 family members, respectively. Of these, only two GH5 proteins, two GH9 proteins, twelve GH16 proteins and three GH17 proteins have been analyzed (Carbohydrate Active Enzymes database (see text footnote 1); Lombard et al., 2014), but did not exhibit β-1,3;1,4-glucanase activity. However, not yet characterized proteins belonging to these GH families may be able to catalyze the hydrolysis of β-1,3/1,4-MLGs and might be involved in the generation of immunogenic MLG oligosaccharides upon microbial attack.

Besides *A. thaliana*, also the crop plants pepper and tomato can detect β-1,3/1,4-MLG oligosaccharides (Rebaque et al., 2021), which suggests that the responsible perception machinery is evolutionary highly conserved among dicot plants. Our study supports this conclusion and goes beyond in demonstrating an invariable intraspecific conservation in the dicot species Arabidopsis as well as potential evolutionary maintenance in the monocot lineage.

Perception of carbohydrate-based ligands was shown to be mediated by LysM domain containing PRRs (Miya et al., 2007; Petutschnig et al., 2010; Shimizu et al., 2010; Willmann et al., 2011; Wan et al., 2012; Cao et al., 2014; Desaki et al., 2018; Mélida et al., 2018; Xue et al., 2019; Wanke et al., 2020). The LysM RLK CERK1 has been suggested as co-receptor for several carbohydrate-based ligands due to its involvement in the perception of chitin, peptidoglycan and β-1,3-glucans in Arabidopsis (Kaku et al., 2006; Miya et al., 2007; Petutschnig et al., 2010; Willmann et al., 2011; Mélida et al., 2018; Wanke et al., 2020). Moreover, the LysM proteins LYK4, LYK5, and LYM2 are components of chitin perception and closure of plasmodesmata in response to chitin, respectively (Kaku et al., 2006; Miya et al., 2007; Petutschnig et al., 2010; Wan et al., 2012; Faulkner et al., 2013; Cao et al., 2014; Erwig et al., 2017; Xue et al., 2019; Cheval et al., 2020). However, our results indicate that detection of β-1,3/1,4-MLG oligosaccharides does not require the LysM proteins CERK1, LYK4, LYK5, and LYM2 in Arabidopsis. Notably, Arabidopsis CERK1 is also not required for the activation of PTI in response to cellobiose (de Azevedo Souza et al., 2017). Whether or not this also holds true for glucose trimers and cellodextrins, which have recently been shown to have comparatively higher elicitor activities (Locci et al., 2019) remains to be shown. However, our data are in conflict with the observations of Rebaque et al. (2021), who suggest that MLG perception is at least partially dependent on CERK1, LYK4, and LYK5. It is hard to rationalize this discrepancy, but the direct comparison of β-1,3/1,4-MLG oligosaccharide-induced MAPK activation in the wildtype and corresponding mutant genotypes that we conducted on the same membrane revealed no significant difference.

We also tested mutants of the prototypical protein MAMP receptors FLS2 and EFR (Gómez-Gómez and Boller, 2000; Zipfel et al., 2006), as well as the promiscuous co-receptor BAK1 and adaptor kinase SOBIR1, which are involved in the perception of many proteinaceous ligands (Chinchilla et al., 2006; Heese et al., 2007; Roux et al., 2011; Schwessinger et al., 2011). In support of a role in protein-derived MAMP/DAMP receptor complex formation, these proteins are dispensable for β-1,3/1,4-MLG oligosaccharide recognition. The observation that known components of the immune system are not implicated in β-1,3/1,4-MLG oligosaccharide perception implies that yet unknown molecular components mediate immune activation in response to β-1,3/1,4-MLG oligosaccharides. In previous studies, elicitor-insensitive Arabidopsis accessions have been used to identify immune receptors and signaling components (Gómez-Gómez et al., 1999; Gómez-Gómez and Boller, 2000; Jehle et al., 2013; Zhang et al., 2013). However, more than 100 *A. thaliana* accessions tested in this study were sensitive to β-1,3/1,4-MLG oligosaccharides indicating that the sensing and signaling machinery is highly conserved. We look forward to the identification of the molecular components required for β-1,3/1,4-MLG oligosaccharide perception by forward genetics in the future.

## Data Availability Statement

The raw data supporting the conclusions of this article will be made available by the authors, without undue reservation.

## Author Contributions

VL, HB, EP, and SB conceived and designed the experiments. SB conducted all experiments, except for HPAEC-PAD, and MALDI-TOF analyses that were performed by GA and NJ. SB, EP, HB, and VL analyzed and discussed the data. SB, EP, and VL wrote the manuscript. All authors contributed to the article and approved the submitted version.

## Conflict of Interest

The authors declare that the research was conducted in the absence of any commercial or financial relationships that could be construed as a potential conflict of interest.

## Footnotes

1 http://www.cazy.org

## Supplementary Material

Supplementary Figure 1 Thin Layer Chromatography (TLC) of enzymatic degradation of the *H. vulgare* β-1,3/1,4-MLG polysaccharide. 10 mg ml^–1^ β-1,3/1,4-MLG polysaccharide of *H. vulgare* were dissolved in 100 mM sodium phosphate buffer (pH = 6.5) and incubated with active lichenase (0.025 U ml^–1^) of *B. subtilis*. Upon 0, 15, 30, 45, 60, 120, and 240 min incubation time, samples were taken and the enzyme was inactivated by incubation for 15 min in boiling water. For TLC, 5 μl of the respective hydrolyzate was applied to the plate. As controls, 5 μl of a mixture of a β-1,3/1,4-MLG tetrasaccharide and trisaccharide (10 mg ml^–1^) as well as β-1,3/1,4-MLG polysaccharide (10 mg ml^–1^) were included. The TLC running buffer contained isopropanol:ethylacetate:H_2_O in a ratio of 2:2:1. The carbohydrates were visualized by wetting the plate with 10% sulfuric acid in methanol and incubation at 99°C for 30–60 min.

Supplementary Figure 2 Verification of masses and HPAEC-PAD profiles of commercially available β-1,3/1,4-MLG MLG oligosaccharides. The purity of the purchased β-1,3/1,4-MLG MLG oligosaccharides was tested with MALDI-TOF **(A,C,E,G)** and HPAEC-PAD **(B,D,F,H)**. For MALDI-TOF, 10 μg ml^–1^ of the respective β-1,3/1,4-MLG oligosaccharide was mixed 1:5 with a 2,5-dihydrobenzoic acid MALDI matrix and analyzed. The expected masses of the pure β-1,3/1,4-MLG oligosaccharides as well as the sodium and potassium adducts are indicated. For HPAEC-PAD analysis, 56 or 45 μM of the MLG tetrasaccharide or MLG trisaccharide, respectively, were analyzed.

Supplementary Figure 3 Thin Layer Chromatography (TLC) of enzymatic degradation of the *H. vulgare* β-1,3/1,4-MLG polysaccharide. 10 mg ml^–1^ β-1,3/1,4-MLG polysaccharide of *H. vulgare* were dissolved in 100 mM Sodium phosphate buffer (pH = 6.5) and incubated with active lichenase (1 U ml^–1^) of *B. subtilis* for 60 min. The enzyme was inactivated by incubating the hydrolyzate for 15 min in boiling water. For TLC, 5 μl of the hydrolyzate was applied to the plate. The TLC running buffer contained isopropanol:ethylacetate:H_2_O in a ratio of 2:2:1. The carbohydrates were visualized by wetting the plate with 10% sulfuric acid in methanol and incubation at 99°C for 30–60 min.

Supplementary Figure 4 Lichenase-generated β-1,3/1,4-MLG oligosaccharides induce phosphorylation of MAPK in various Arabidopsis accessions. MAPK activation in 14-day-old seedlings of different *A. thaliana* ecotypes in response to β-1,3/1,4-MLG oligosaccharides. β-1,3/1,4-MLG oligosaccharides were generated upon incubation of the β-1,3/1,4-MLG polysaccharide (10 mg ml^–1^) with the *B. subtilis* lichenase (1 U ml^–1^) for 60 min (Supplementary Figure 3). For the treatment, a 1:10 dilution of the hydrolysis products was used. Sodium phosphate buffer (1 mM) served as negative control. As loading control, the PVDF membrane was stained with Coomassie Brilliant Blue (CBB). Data show the result from one biological replicate.
