# Supplementary figures and images for: Mixed Linkage β-1,3/1,4-Glucan Oligosaccharides Induce Defense Responses in Hordeum vulgare and Arabidopsis thaliana
Source: Front Plant Sci. 2021 Jun 17;12:682439. doi: 10.3389/fpls.2021.682439 (PMC8247929; doi:10.3389/fpls.2021.682439)

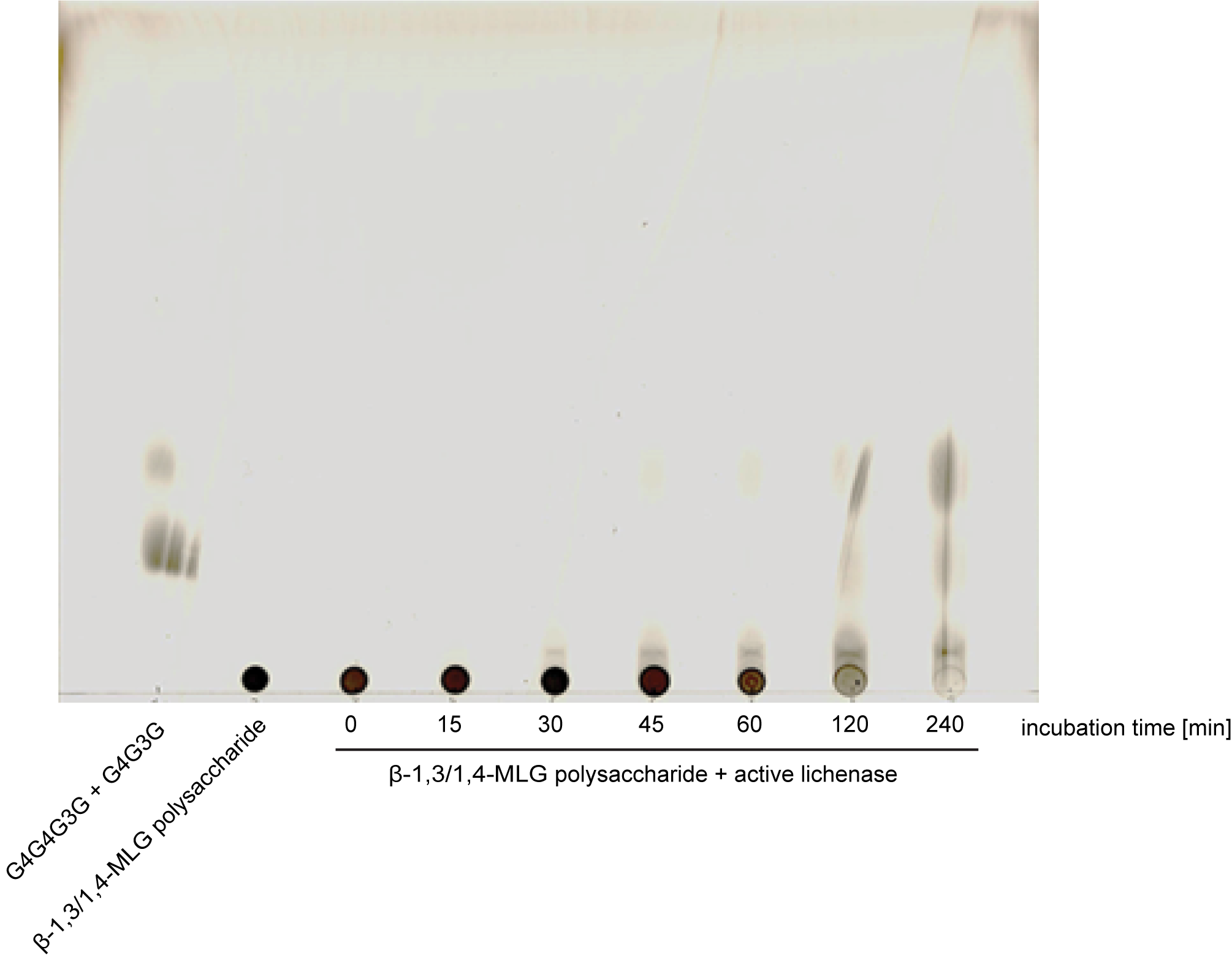

Supplement: Supplementary Figure 1 — Thin Layer Chromatography (TLC) of enzymatic degradation of the H. vulgare β-1,3/1,4-MLG polysaccharide. 10 mg ml–1 β-1,3/1,4-MLG polysaccharide of H. vulgare were dissolved in 100 mM sodium phosphate buffer (pH = 6.5) and incubated with active lichenase (0.025 U ml–1) of B. subtilis. Upon 0, 15, 30, 45, 60, 120, and 240 min incubation time, samples were taken and the enzyme was inactivated by incubation for 15 min in boiling water. For TLC, 5 μl of the respective hydrolyzate was applied to the plate. As controls, 5 μl of a mixture of a β-1,3/1,4-MLG tetrasaccharide and trisaccharide (10 mg ml–1) as well as β-1,3/1,4-MLG polysaccharide (10 mg ml–1) were included. The TLC running buffer contained isopropanol:ethylacetate:H2O in a ratio of 2:2:1. The carbohydrates were visualized by wetting the plate with 10% sulfuric acid in methanol and incubation at 99°C for 30–60 min. [file Image_1.TIF]

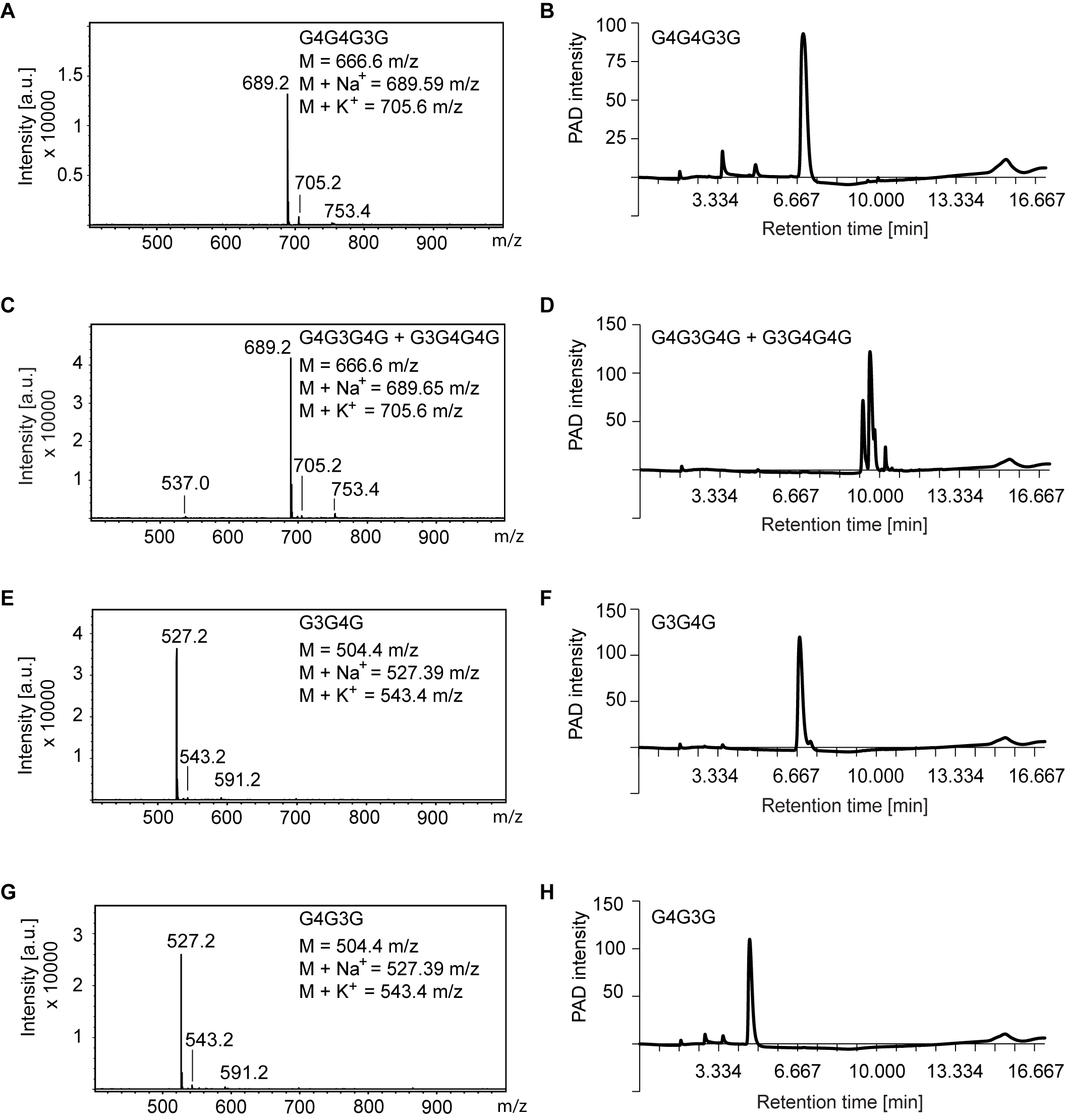

Supplement: Supplementary Figure 2 — Verification of masses and HPAEC-PAD profiles of commercially available β-1,3/1,4-MLG MLG oligosaccharides. The purity of the purchased β-1,3/1,4-MLG MLG oligosaccharides was tested with MALDI-TOF (A,C,E,G) and HPAEC-PAD (B,D,F,H). For MALDI-TOF, 10 μg ml–1 of the respective β-1,3/1,4-MLG oligosaccharide was mixed 1:5 with a 2,5-dihydrobenzoic acid MALDI matrix and analyzed. The expected masses of the pure β-1,3/1,4-MLG oligosaccharides as well as the sodium and potassium adducts are indicated. For HPAEC-PAD analysis, 56 or 45 μM of the MLG tetrasaccharide or MLG trisaccharide, respectively, were analyzed. [file Image_2.TIF]

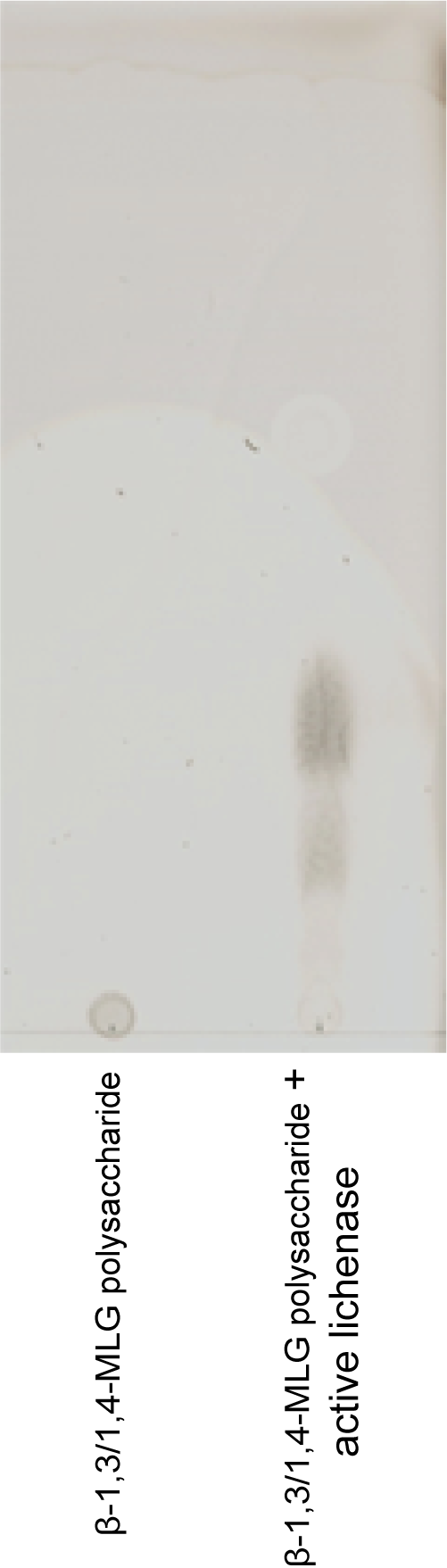

Supplement: Supplementary Figure 3 — Thin Layer Chromatography (TLC) of enzymatic degradation of the H. vulgare β-1,3/1,4-MLG polysaccharide. 10 mg ml–1 β-1,3/1,4-MLG polysaccharide of H. vulgare were dissolved in 100 mM Sodium phosphate buffer (pH = 6.5) and incubated with active lichenase (1 U ml–1) of B. subtilis for 60 min. The enzyme was inactivated by incubating the hydrolyzate for 15 min in boiling water. For TLC, 5 μl of the hydrolyzate was applied to the plate. The TLC running buffer contained isopropanol:ethylacetate:H2O in a ratio of 2:2:1. The carbohydrates were visualized by wetting the plate with 10% sulfuric acid in methanol and incubation at 99°C for 30–60 min. [file Image_3.TIF]

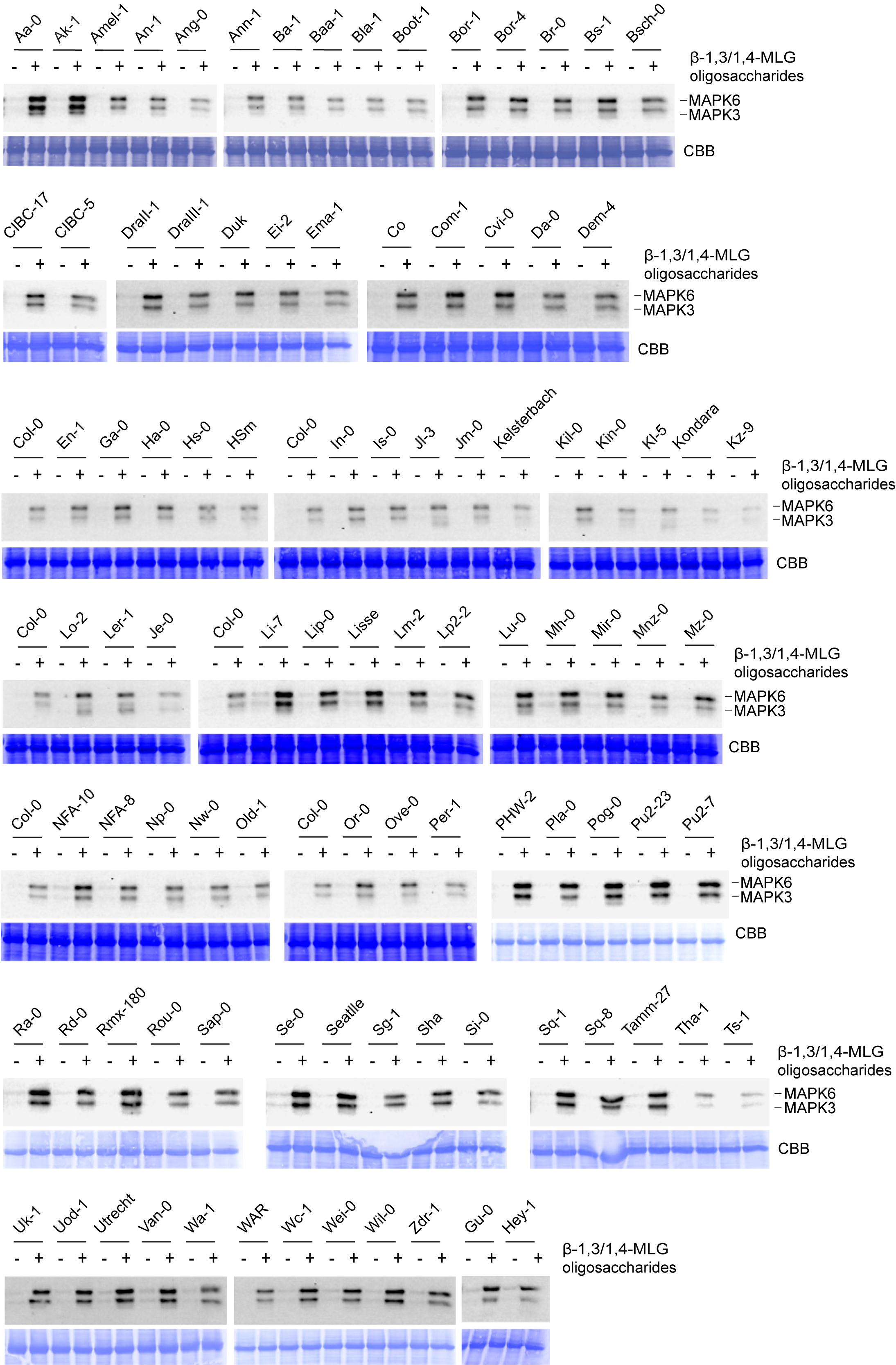

Supplement: Supplementary Figure 4 — Lichenase-generated β-1,3/1,4-MLG oligosaccharides induce phosphorylation of MAPK in various Arabidopsis accessions. MAPK activation in 14-day-old seedlings of different A. thaliana ecotypes in response to β-1,3/1,4-MLG oligosaccharides. β-1,3/1,4-MLG oligosaccharides were generated upon incubation of the β-1,3/1,4-MLG polysaccharide (10 mg ml–1) with the B. subtilis lichenase (1 U ml–1) for 60 min (Supplementary Figure 3). For the treatment, a 1:10 dilution of the hydrolysis products was used. Sodium phosphate buffer (1 mM) served as negative control. As loading control, the PVDF membrane was stained with Coomassie Brilliant Blue (CBB). Data show the result from one biological replicate. [file Image_4.TIF]
