# Supplementary material for: Mixed Linkage β-1,3/1,4-Glucan Oligosaccharides Induce Defense Responses in Hordeum vulgare and Arabidopsis thaliana
Source: Front Plant Sci. 2021 Jun 17;12:682439. doi: 10.3389/fpls.2021.682439 (PMC8247929; doi:10.3389/fpls.2021.682439)
Supplement: Supplementary file 5 [file Table_1.DOCX]

**Supplementary Table 1**. Arabidopsis accessions used in this work.

| **Name** | **Abbreviation** | **NASC ID** | **GWAS-ID** |
| --- | --- | --- | --- |
| Aua/Rhon | Aa-0 | - | 700 |
| Achkarren | Ak-1 | - | 6987 |
| Ameland-Firehouse | Amel-1 | - | 6990 |
| Antwerpen | An-1 | - | 6898 |
| Angleur | Ang-0 | - | 6992 |
| Annecy | Ann-1 | - | 6994 |
| Blackmount | Ba-1 | - | 7014 |
| Baarlo | Baa-1 | - | 7002 |
| Blanes | Bla-1 | - | 7015 |
| Boot, Eksdale | Boot-1 | - | 7026 |
| Borky | Bor-1 | - | 5837 |
| Borky | Bor-4 | - | 6903 |
| Br | Br-0 | - | 6904 |
| Basel | Bs-1 | - | 8270 |
| Busch | Bsch-0 | - | 7031 |
| Burren | Bur-0 | N102 | - |
| Canary Island | Can-0 | N1064 | - |
| CIBC | CIBC-17 | - | 6907 |
| CIBC | CIBC-5 | - | 6730 |
| Coimbra | Co | - | 7081 |
| Columbia-0 | Col-0 | J. Dangl, University of North Carolina, USA. | - |
| Compiegne | Com-1 | - | 7092 |
| Catania | Ct-1 | N1094 | - |
| Cape Verde Islands | Cvi-0 | - | 6911 |
| Darmstadt | Da-0 | - | 7094 |
| - | Dem-4 | - | 8233 |
| - | DraII-1 | - | 8284 |
| - | DraIII-1 | - | 8285 |
| - | Duk | - | 6008 |
| Edinburgh | Edi-0 | N1122 | - |
| Eifel | Ei-2 | - | 6915 |
| East Malling | Ema-1 | - | 5736 |
| Enkheim | En-1 | - | 8290 |
| Gabelstein | Ga-0 | - | 6919 |
| Gueckingen | Gu-0 | - | 6922 |
| Hannover | Ha-0 | - | 7163 |
| Heythuysen | Hey-1 | - | 7166 |
| Hilversum | Hi-0 | - | 8304 |
| Hannover/Stroehen | Hs-0 | - | 8310 |
| Horni Smrcne | HSm | - | 8236 |
| Isenburg | In-0 | - | 8311 |
| Isenburg | Is-0 | - | 8312 |
| Jena | Je-0 | - | 7181 |
| Jl | Jl-3 | - | 7424 |
| Jamolice | Jm-0 | - | 8313 |
| Kelsterbach | Kelsterbach | - | 8420 |
| Killean | Kil-0 | - | 7192 |
| Kindalville | Kin-0 | - | 6926 |
| Köln | Kl-5 | - | 7199 |
| Kaunas | Kn-0 | N1286 | - |
| Kondara | Kondara | - | 6929 |
| Kazakhstan | Kz-9 | - | 6931 |
| Landsberg | Ler-1 | - | 6932 |
| Limburg | Li-7 | - | 7231 |
| Lipowiec | Lip-0 | N1136 | - |
| - | Lisse | - | 8430 |
| Le Mans | Lm-2 | - | 8329 |
| Lorrach | Lo-2 |  | 7242 |
| Lipovec | Lp2-2 | - | 7520 |
| Lund | Lu-0 | - | 8334 |
| Mühlen | Mh-0 | N1368 | - |
| Miramare | Mir-0 | - | 8337 |
| Mainz | Mnz-0 | - | 7244 |
| Martuba | Mt-0 | N1380 | - |
| Merzhausen | Mz-0 | - | 6940 |
| NFA | NFA-10 | - | 6943 |
| NFA | NFA-8 | - | 6944 |
| Nossen | No-0 | N77128 | - |
| Nieps | Np-0 | - | 7268 |
| Neuweilnau | Nw-0 | - | 8348 |
| Oldenburg | Old-1 | - | 7280 |
| Oranienstein | Or-0 | - | 7282 |
| Ovelgoenne | Ove-0 | - | 7287 |
| Oystese | Oy-0 | N1436 | - |
| Perm | Per-1 | - | 8354 |
| - | PHW-2 | - | 8243 |
| Playa de Aro | Pla-0 | - | 7300 |
| Poppelsdorf | Po-0 | N1470 | - |
| Point Grey | Pog-0 | - | 7306 |
| Prudka | Pu2-23 | - | 6951 |
| Prudka | Pu2-7 | - | 6956 |
| Randan | Ra-0 | - | 6958 |
| Rodenbach | Rd-0 | - | 8366 |
| St. Josephs | Rmx-180 | - | 7525 |
| Rouen | Rou-0 | - | 7320 |
| Rschew | Rsch-4 | N1494 | - |
| Slapy | Sap-0 | - | 8378 |
| San Eleno | Se-0 | - | 6961 |
| Seatlle | Seatlle-0 | - | 8245 |
| San Feliu | Sf-2 | N1516 | - |
| St. Georgen | Sg-1 | - | 7344 |
| Pamiro-Alay | Sha | - | 6962 |
| Siegen | Si-0 | - | 7337 |
| Sq | Sq-1 | - | 6966 |
| Sq | Sq-8 | - | 6967 |
| Tammisari | Tamm-27 | - | 6969 |
| The Hague | Tha-1 |  | 7353 |
| Tossa de Mar | Ts-1 | - | 6970 |
| Tsu | Tsu-0 | N1564 | - |
| Umkirch | Uk-1 | - | 7378 |
| Uod | Uod-1 | - | 6975 |
| Utrecht | Utrecht | - | 7382 |
| Vancouver | Van-0 | - | 6977 |
| Warschau | Wa-1 | - | 6978 |
| - | WAR | - | 7477 |
| Westercelle | Wc-1 | - | 7404 |
| Weiningen | Wei-0 | - | 6979 |
| Wilna | Wil-0 | - | 0 |
| Wilna | Wil-2 | N1596 | - |
| Wassilewskija | Ws-0 | - | - |
| Wü | Wu-0 | N6195 | - |
| Zdarec | Zdr-1 | - | 6984 |
| Zurich | Zu-0 | N1626 | - |
