# Supplementary material for: Mixed Linkage β-1,3/1,4-Glucan Oligosaccharides Induce Defense Responses in Hordeum vulgare and Arabidopsis thaliana
Source: Front Plant Sci. 2021 Jun 17;12:682439. doi: 10.3389/fpls.2021.682439 (PMC8247929; doi:10.3389/fpls.2021.682439)
Supplement: Supplementary file 6 [file Table_2.docx]

**Supplementary Table 2**. Oligonucleotides used for qRT-PCRs.

| **Primer** | **Sequence** | **Purpose** | **Primer Melting Temperature (T_m_) [°C]** |
| --- | --- | --- | --- |
| DS64 | GACGCTTCATCTCGTCC | qRT PCR of *UBIQUITIN5* | 59 |
| DS65 | GTAAACGTAGGTGAGTCCA | qRT PCR of *UBIQUITIN5* | 58 |
| JE73 | GGTCACAACAATCCGGAAGA | qRT PCR of *WRKY33* (from Cao et al., 2014) | 62 |
| JE74 | GGAGAGACAAGAGAAGGAGAGA | qRT PCR of *WRKY33* (from Cao et al., 2014) | 62 |
| JE79 | TCACCGAGCGTACAACTTATTCC | qRT PCR of *WRKY53* (from Cao et al., 2014) | 64 |
| JE80 | CGTTTATCGATGCCGGAGATT | qRT PCR of *WRKY53* (from Cao et al., 2014) | 62 |
